# Coronectomy versus surgical removal of the lower third molars with a high risk of injury to the inferior alveolar nerve. A bibliographical review

**DOI:** 10.4317/medoral.20432

**Published:** 2015-04-10

**Authors:** Javier Moreno-Vicente, Rocío Schiavone-Mussano, Enrique Clemente-Salas, Antoni Marí-Roig, Enric Jané-Salas, José López-López

**Affiliations:** 1 DDS. Dentistry. Postgraduate student of Oral Medicine, Oral Surgery and Implantology. Dental School. University of Barcelona, Catalonia, Spain; 2 DDS. Medical specialist in stomatology. Professor of Master in Oral Medicine, Oral Surgery and Implantology. Dental School, University of Barcelona, Catalonia, Spain; 3 PhD. DDS. MD. Oral and Maxillofacial Surgery. Head of Service. Bellvitge University Hospital (HUB), Catalonia, Spain. Professor and Manage Master of Oral Medicine, Oral Surgery and Implantology. Dental School, University of Barcelona, Catalonia, Spain; 4 PhD. DDS. MD. Medical specialist in stomatology. Professor of Oral Pathology. Dental School, University of Barcelona, Catalonia, Spain

## Abstract

**Background:**

Coronectomy is the surgical removal of the crown of the tooth deliberately leaving part of its roots. This is done with the hope of eliminating the pathology caused, and since the roots are still intact, the integrity of the inferior alveolar nerve is preserved.

**Objectives:**

The aim is to carry out a systematic review in order to be able to provide results and conclusions with the greatest scientific evidence possible.

**Material and Methods:**

A literature review is carried out through the following search engines: Pubmed MEDLINE, Scielo, Cochrane library and EMI. The level of evidence criteria from the Agency for Healthcare Research and Quality was applied, and the clinical trials’ level of quality was analyzed by means of the JADAD criteria.

**Results:**

The following articles were obtained which represents a total of 17: 1 systematic review, 2 randomized clinical trials and 2 non-randomized clinical trials, 3 cohort studies, 2 retrospective studies, 3 case studies and 4 literature reviews.

**Conclusions:**

Coronectomy is an adequate preventative technique in protecting the inferior alveolar nerve, which is an alternative to the conventional extraction of third molars, which unlike the former technique, presents a high risk of injury to the inferior alveolar nerve. However, there is a need for new clinical studies, with a greater number of samples and with a longer follow-up period in order to detect potential adverse effects of the retained roots.

**Key words:** Coronectomy, inferior alveolar nerve, nerve injury, wisdom tooth removal, paresthesia, and systematic review.

## Introduction

The evolution of the human being has entailed different changes in biology. Among many others, it has increased the incidence of dental impactions. In daily practice, the impacted third molar are a frequent occurrence, which oscillates between different studies, and according to Long H et al., its frequency is between 35.9% and 58.7% (1). These impacted molars imply associated pathology which is well-documented: cysts, tumors, cavities and pericoronitis, are among the most frequent pathologies. Therefore, extraction is the appropriate treatment in the majority of these cases. However, the surgical procedure is not free of complications; among which the following can be highlighted: injury and nerve disorders, pain, infection and dry socket, along with other complications (2). Injury to the inferior alveolar nerve (IAN) during the third molar surgery, entails a sensory deficit that may be temporary (from 0.41% to 8.1%) or permanent (from 0.0145 to 3.6%), which depending on the patient can contain a serious problem (3-7). We know of different radiographic risk indicators which are useful in evaluating the risk of injuring the IAN during surgery. These are signs that can increase the risk of nerve injury by up to 35.64% when present (6-9).

There are numerous alternative techniques described in the literature in order to minimize this risk, among them is the coronectomy, first described in 1984 by Ecuyer and Debien (10). The coronectomy or intentional partial odontectomy, is the removal of the crown of the lower third molar, deliberately leaving part of its root or roots in the jaw, and as mentioned by Sencimen M et al. without posterior pulp treatment (9). Thus the pathology caused by pericoronitis is eliminated, seeing as we are able to achieve direct closure of the wound and the roots remain intact, therefore preserving the integrity of the IAN (5,7,11). While the objective of this technique is very clear, it is not free of controversy. The surgeon should evaluate the possibility of an infectious complication of pulpal and/or periodontal origin. If a pulp infection, or eruption of the roots (usually within a few months) arise, a second surgery is necessary to complete the extraction (12). In this case, those in favor of the technique indicate that there is less risk of injury to the IAN since generally a root migration occurs and it is no longer close (1).

Based on the above, we hypothesized: is coronectomy a useful technique in oral surgery? And our aim is to make a systematic review of the literature regarding the utility of this technique.

## Material and Methods

We performed a review of the published literature related to this topic found in the search engines Pubmed MEDLINE, Scielo, Cochrane library and Índice Médico Español (IME) (Spanish Medical Index) with the following key words: “coronectomy,” “coronectomy AND oral surgery,” “coronectomy AND third molar,” “coronectomy AND dentistry,” “coronectomy AND dental treatment,” and “intentional partial odontoectomy” using each independently from the other. We included articles published in the last 10 years, and we did not apply restrictions of languages or other exclusion criteria to the search.

After obtaining articles through this strategy, the classification of the recommendations was applied to each one of the articles, by two independent authors, based on the level of scientific evidence available according to the Agency for Healthcare Research and Quality (13), and we included those that were classified within the type I (A and B), II (A and B) and III.

After these exclusion criteria were applied, we began to analyze the selected articles and we classified them; in the case of a clinical study, it was classified depending on its level of quality, applying the JADAD criteria (14).

## Results

**Figure 1:**
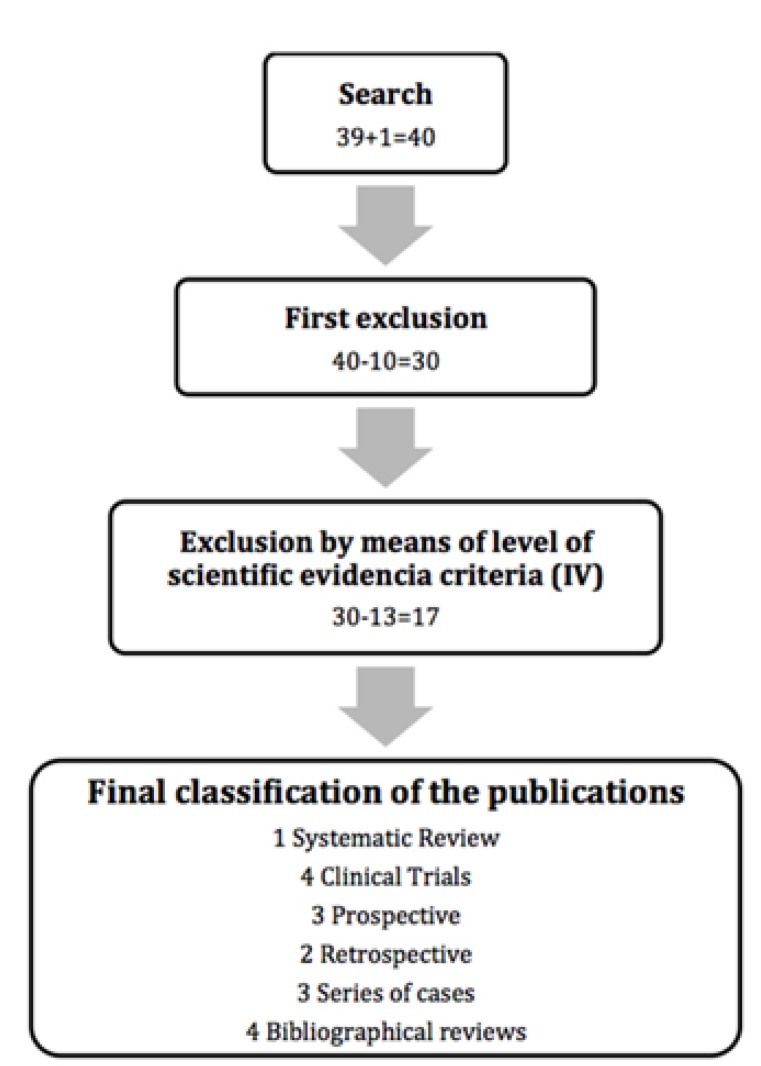
Diagram with the selection criteria for the chosen publications.

By searching Pubmed MEDLINE, Scielo, Cochrane library and Índice Médico Español (IME) we obtained a total of 40 articles. In Scielo only one publication was found with the key word “coronectomy,” in the IME and Cochrane library there were no articles found using the previous key words, while in Pubmed MEDLINE, 188 publications were found that can be broken down as follows: “Coronectomy” (48 articles), “coronectomy AND oral surgery” (44 articles), “coronectomy AND third molar” (43 articles), “coronectomy AND dentistry,” (38 articles) “coronectomy and dental treatment” (10 articles) and “intentional partial odontectomy” (5 articles). Once all of the articles were cross-checked, there were a total of 39 articles. Of the 40 resulting articles (39 from Pubmed and 1 from Scielo), 10 were ruled out at first for not being of interest to our review, thus leaving us with 30 (Fig. 1).

The criteria for determining the level of scientific evidence from the Agency for Healthcare Research and Quality (13) was applied to the 30 resulting articles. After this we exclude 13 publications since they did not meet our inclusion criteria (level of evidence Ia, Ib, IIa, IIb, III), seeing as they were presentations of an isolated clinical case or consisted of opinions, with a low level of evidence. We applied the JADAD criteria (14) to the 17 remaining articles, in order to analyze their level of quality ( Table 1).

**Table 1:**
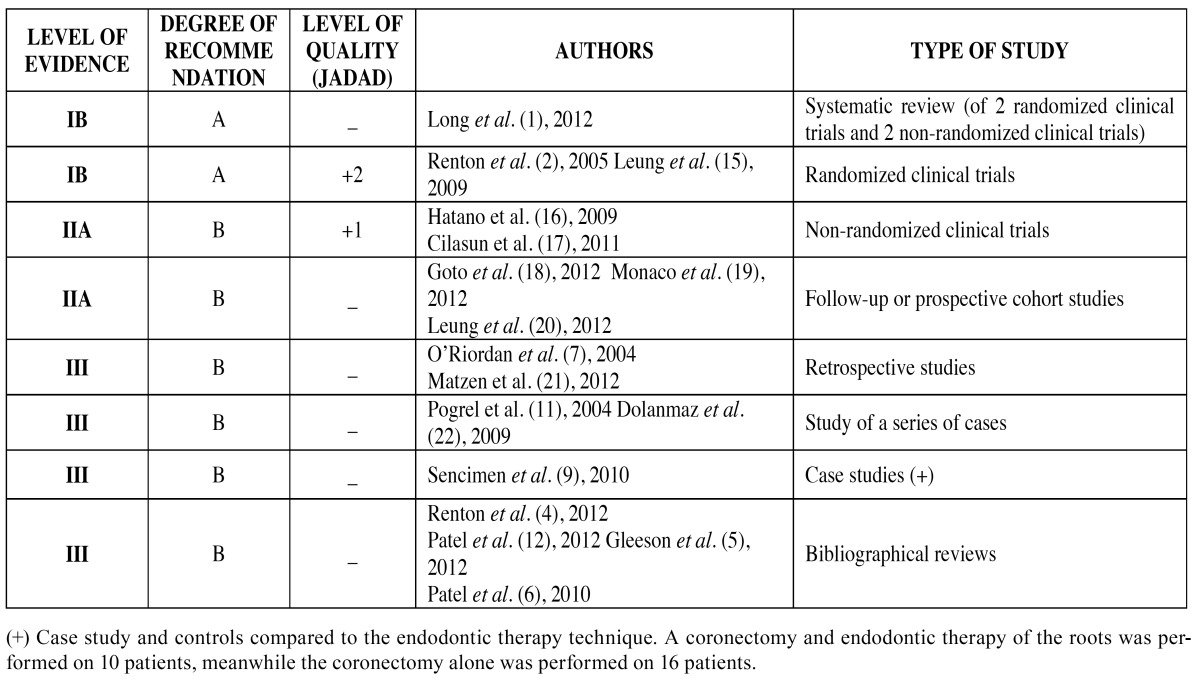
Classification of the studies selected for the review according to type of study, level of evidence and degree of recommendation and level of quality.

We only obtained one systematic review that was published in 2012, based on two randomized clinical trials and two nonrandomized ones. We included these four studies separately in the review; the study by Renton *et al*. (2) in 2005 y Leung *et al*. (15) in 2009 which was one of these randomized clinical studies, and Hatano *et al*. (16) in 2009 and Cilasun *et al*. (17) which was one of the nonrandomized clinical studies.

Also included in the revision are 3 cohort or prospective studies, all of which were published in 2012. They are the studies performed by Goto *et al*. (18), in which a clinical follow-up was carried out, with CT at 12 months after the coronectomy; Monaco *et al*. (19), in which the postoperative complications were assessed with a maximum follow-up of 12 months; and Leung *et al*. (20) in which the results of their original study were published with a follow-up period of 6 years.

The rest of articles that we analyzed include retrospective studies, as is the case of O’Riordan *et al*. (6), and clinical studies with few series of cases, which we consider appropriate to include due to the little amount of published clinical studies.

Finally, we included 5 bibliographic reviews, those of Patel *et al*. (6,12) in 2010 y 2012, Gleeson *et al*. (5) in 2012 y Renton *et al*. (4) in 2012. Additionally, articles were included that were not directly related with the coronectomy technique but allowed us to define the problem of the third molar surgery, as well as its complications.

## Discussion

In order to analyze the various articles found, we have organized the discussion based on the following sections: (i) Patient Selection and Diagnostic Method, (ii) Study Population, (iii) Surgical Technique, (iv) Variables Analyzed, (v) Technique Results and (vi) Follow-up.

(i) Patient Selection and Diagnostic Method

Among the different authors who have studied this technique, the diagnosis is radiologically based on the orthopantomograph, periapical and/or mandibular CT scan.

Traditionally for these types of interventions the panoramic x-ray is the first method of choice, seeing as it can be combined with intraoral images of different projections (21). Using the orthopantomograph we can see radiographic signs which are indicative that the IAN is possibly at risk, among which we can distinguish the darkening, deflection and narrowing of the root, and diversion, narrowing and interruption of the dental canal (2,4,7,8). Recent studies conclude that despite the absence of these signs, we cannot ensure the existence of the direct contact with the mandibular canal. The three most valid signs are the interruption and diversion of the canal, and the darkening of the roots (9), although for Céspedes *et al*. the only valid signs are the interruption of the canal and the darkening of the roots (8). With the evolution of new technologies, the latest trials incorporate the CT mandibular study (9,16-19,21).

In a recent work published by Matzen *et al*. (21) the objective was to evaluate, through case studies and controls, the influence of the CBCT (Cone beam CT) on the decision-making process in the preoperative period of a lower third molar, as well as to identify the radiographic factors with a greater impact on the surgical decision of coronectomy or complete extraction. The author selected a sample of 186 third molars with suspicion of high risk of injury to the IAN based on the panoramic x-ray, and he carried out a CBCT on them.

In the results, a 12% change of opinion was observed in those clinicians who had seen the CBCT, meanwhile the other 88% agreed on the first treatment plan (with the orthopantomography) and the second (with the CBCT). Additionally, in the logistic regression it was confirmed that among all of the registered variables, the most significant ones for deciding in favor of the coronectomy technique were the direct contact between the roots and the mandibular canal (OR of 101.8, *p*<0.001 ) and the flexion of the distal root (oral position) (OR of 23.3 p=0.002). Also, among the molars in direct contact, those that underwent the coronectomy, it was observed that the probability of choosing this technique was very high if the lumen of the channel narrowed, up to 40 times higher if there was flexion (oral) of the distal root and 33 times higher if the canal already positioned itself in flexion or opening of the roots (21).

(ii) Study Population

The largest group of patients was the group presented by Leung *et al*. (15). They selected 349 lower molars, candidates for a coronectomy. They divided the sample into two groups, and they performed the coronectomy on 171 wisdom teeth. Another large group of interventions was presented in the work of Hatano *et al*. (16), with 102 patients treated with a coronectomy and the respective control group. It was followed by Renton *et al*. (2), with 94 wisdom teeth as the sample in the coronectomy group, and finally Cilasun *et al*. (17), with a sample of 88 coronectomies. It should be noted that the sample for Cilasun *et al*. (17), Leung *et al*. (15), and Renton *et al*. (2), was based on the number of wisdom teeth, while Hatano *et al*. (16), based it on the number of patients, without indicating the number of lower third molars on which they operated.

The rest of studies that strictly evaluate the technique, do not include a control group, as is the case of Goto *et al*. (18) who performed a coronectomy on 116 lower third molars, Monaco *et al*. (19) on 43, O’Riordan *et al*. (7) on 52, Dolanmaz *et al*. (22) on 47 y Pogrel *et al*. (11) on 50. ( Table 2).

**Table 2:**
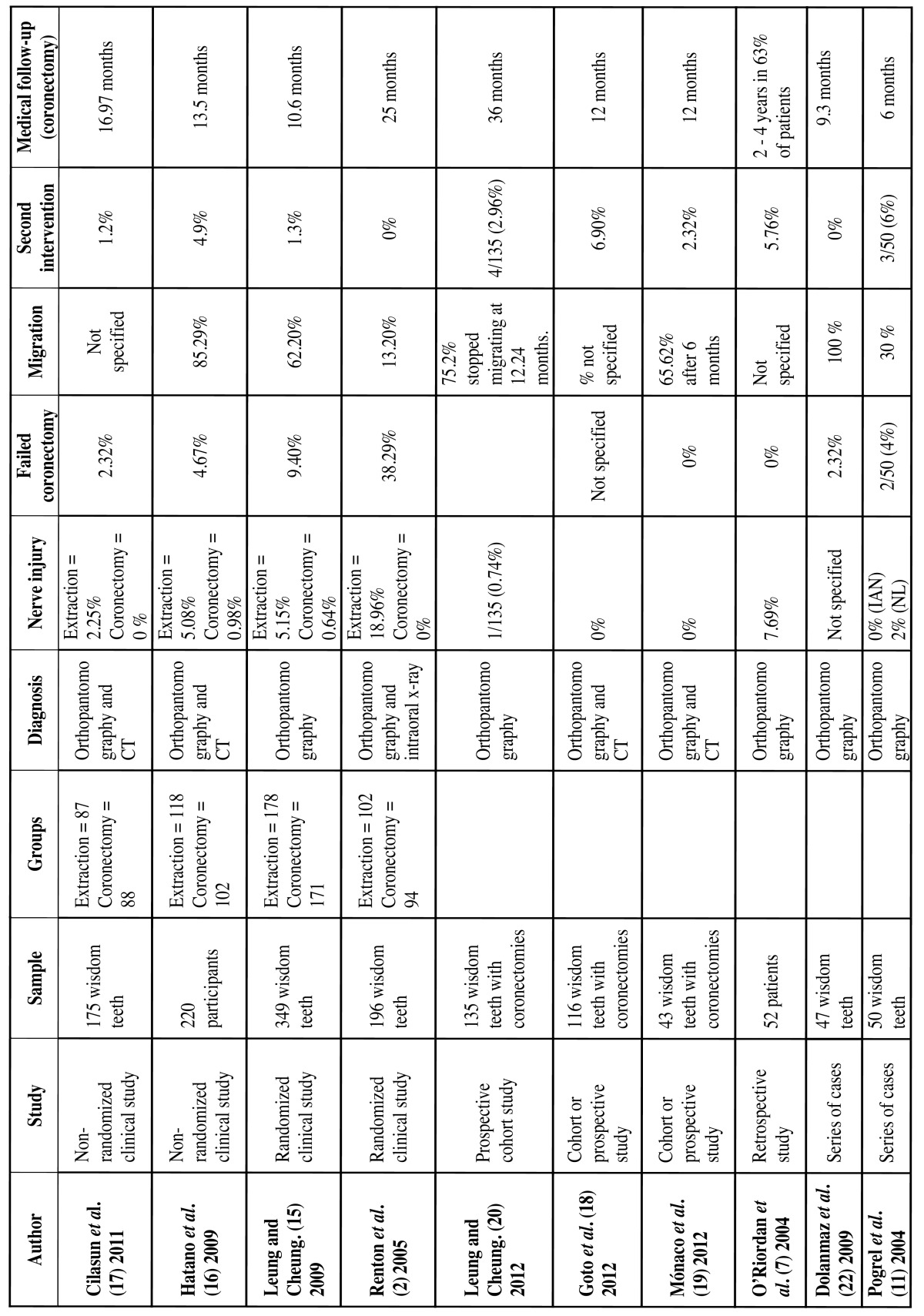
Most relevant clinical data from the selected studies in our review.

(iii) Surgical Technique

**Figure 2:**
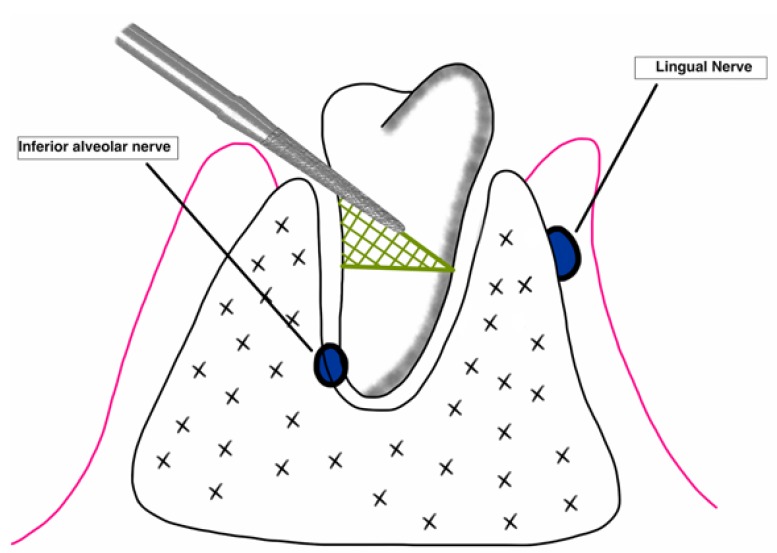
Surgical technique (coronectomy). Compilation prepared by the authors, based on the description from Pogret et al. (11). We can see that the drill is placed at 45º from the vestibular to the lingual flap with the objective of obtaining a lingual cutting surface of a minimum of 3 mm below the bone level.

The surgical technique described by Pogrel *et al*. (11) consists of raise the vestibular mucoperiosteal flap and the lingual flap. An adequate lingual retractor is placed to prevent injury to the lingual nerve. Subsequently the odontosection is carried out with the contra-angle and a dental drill, and a cutting angle of approximately 45°, in order to obtain a lingual cutting surface of at least 3 mm below the bone margin. The odontosection is performed entirely with the drill in order to minimize the risk of mobilizing the retained roots. However, other authors (7,15,17,19) believe that it is more desirable to complete the odontosection by using a forceps instrument, in order to minimize the risk of injuring the lingual nerve. Subsequently, the surgical wound is irrigated with sterile serum, without performing any pulpal treatment, and sutured in the usual way (Fig. 2).

Only Sencimen *et al*. (9), analyzed the possible effectiveness of the endodontic treatment in the coronectomy procedure. They compared the coronectomy technique described with the coronectomy and pulpar treatment of the root canal with MTA. They concluded that the pulpar treatment is not recommended, due to the fact that it increases the rate of complications and infections that require a second intervention (9). Additionally, it was found that upon removing the roots that were the cause of complications, those that were endodontically treated had not migrated in comparison to the control group, therefore the extraction temporarily damaged the IAN (9). Based on this study, and in spite of having a low sample (16 lower third molars), there is no medical evidence to support the desirability of performing endodontic therapy of the retained roots.

In regard to the use of both pre- and postoperative antibiotic therapy, there is a lot of controversy among the different studies. After analyzing the variables of infection and postoperative pain, there is heterogeneity among the various studies that prevents them from reaching clear conclusions, thanks to the use of antibiotics (1).

(iv) Variables Analyzed

The main variable that was analyzed in all the studies is injury to the IAN and its subtypes. However, not all of the studies described what type of nerve injury was present or the type of diagnostic method that was used. Secondly, postoperative pain (using visual analog scale - VAS), infection and dry socket were analyzed.

In the review that we are hereby presenting, we note that only Monaco *et al*. (19) and Goto *et al*. (18), analyzed the recovery of the second distal molar after the coronectomy, and no study assessed trismus as a postoperative complication.

If we concentrate on the adverse effects of the technique, the intraoperative failure due to accidental mobilization of the roots is assessed, as well as the migration of the roots and the possibility that such migration requires the second intervention. As for the calculation of the root migration among the different studies, it is carried out by means of a few lines on the panoramic or periapical radiograph with a millimeter ruler.

**Figure 3:**
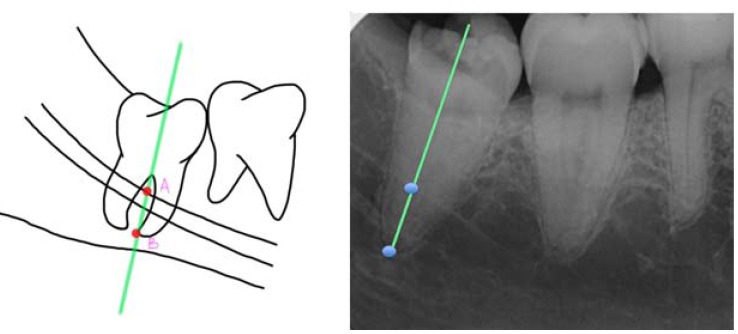
a) Calculation of the root migration through the distance between point A (superior cortical of the mandibular canal) and point B (apical point of lower third molar). Compilation prepared by the authors, based on the technique proposed by Leung *et al*. (15). (b) Example in the form of a radiographic image.

**Figure 4:**
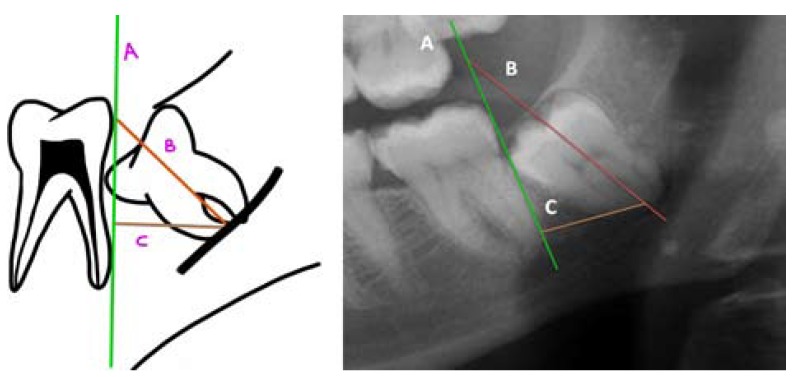
a) Calculation of the root migration according to the technique proposed by Goto *et al*. (18). Lines A (tangential to the second distal molar), B (center of third molar) and C (perpendicular to A and which goes in direction of the apical point of the lower third molar) are traced. Compilation prepared by the authors, based on the technique proposed by Goto *et al*. (18). (b) Example in the form of a radiographic image.

**Figure 5:**
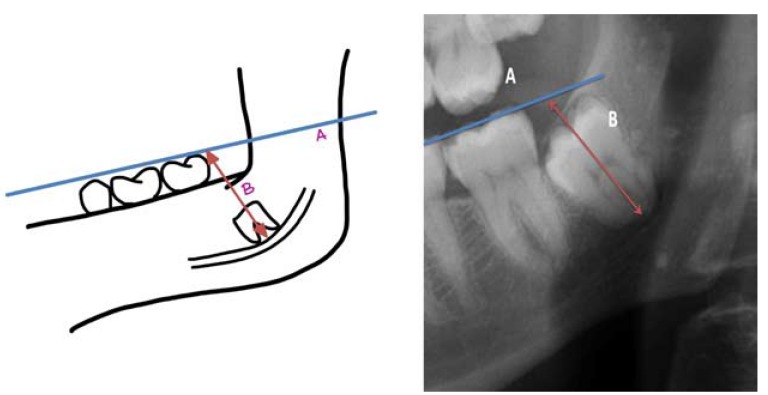
(a) Calculation of the root migration through the distance between line A (occlusal surface of lower molars) and B (longitudinal from the center of the molar up to A). Compilation prepared by the authors, based on the technique proposed by Dolanmaz *et al*. (22). (b) Example in the form of a radiographic image.

Authors such as Leung *et al*. (15) and Monaco *et al*. (19) used the measurement between two points (A and B). One point was marked on the intersection between the highest point of the upper or lower cortical of the mandibular canal and the longitudinal axis of the root, and another on the most apical point of the root (15,19,20) (Fig. 3). On the other hand, Goto *et al*. (18) preferred viewing it by means of the drawing of 3 lines (one that was tangential to the distal part of the lower second molar, another in the center of the lower third molar between the mesial and distal roots, and the last one was perpendicular and connected the first with the center at the apical level of the lower third molar) (18) (Fig. 4). Finally, Dolanmaz *et al*. (22) used the measurement of the intersection between a line that joined the occlusal surfaces of the lower molars and extended to the ramus of the mandible and a longitudinal line in the middle of the roots of the lower third molar (22) (Fig. 5).

(v) Technique Results

We believe that the results found in the systematic review (level of evidence Ib) published by Long *et al*. (1) are interesting, since it included the 4 studies with the greatest scientific evidence with respect to the coronectomy technique ( Renton *et al*. (2), Leung *et al*. (15), Hatano *et al*. (16) and Cilasun *et al*. (17). In the rest of studies, either due to their low number of samples, or due to their brief long-term follow-up or the absence of a control group, it is not possible to extrapolate the results.

Based on the results of Long *et al*. (1), the injury of the inferior alveolar nerve was significantly lower in the coronectomy group (RR of 0.11), whereas the postoperative infection (RR of 1.03), the dry socket (RR of 0.55) and postoperative pain (RR of 1.14) were statistically similar in both groups (1).

If we focus on the complications of a coronectomy (failures of the coronectomy) there is a controversy among different studies, but it is suggested that there are 2 risk factors: the narrowing of the roots and a pattern of vertical retention. The rate of second intervention due to migration (13.2 to 85.29%), exposure of the roots (0 to 1.3%) and/or infection, is relatively low (between 0 to 4.9%) (1).

The average migration distance of the roots at the 2 year mark is approximately 3mm, therefore if the extraction is necessary; the potential risk of nerve injury is significantly reduced (1). However, authors such as Monaco *et al*. (19) in their study with a sample of 43 coronectomies, confirmed that if we compare the migration at the 6 month mark and the 12 month mark, there is no additional migration when measured the second time (19). Authors such as Goto *et al*. (18) in their study with a sample carried out on 101 patients, suggested that the migration is greater in the women (*p* = 0.034), in the age group of <20 years (*p* = 0.004) and conical roots (*p*= 0.007) (18).

In regard to the periodontal damage of the lower second molar, as we have previously described, it has only been studied by two authors. Monaco *et al*. (19), through their cohort study with a sample of 43 coronectomies, they concluded that in almost all of their cases, the root migration determined the bone regeneration on the bone defect distal to the second molar, similar to the migration obtained through orthodontic extrusion (19). On the other hand, Goto *et al*. (18), assessed only the soft tissue at the 12 month mark of follow-up, and they concluded that in 99.2% of the cases the periodontal status distal to the second molar was healthy and did not show inflammation, but neither the periodontal status nor the bone tissue were compared with the preoperative state (18).

In the prospective study of Leung *et al*. (20), they concluded that coronectomy of the lower third molar was safe during the 3 year postoperative follow-up. Among their results, they reported 4.4% of infection during the first 7 postoperative days, 43% of pain during the immediate postoperative period, 0% of dry socket, 0% of lingual nerve injury and only 1 case of paresthesia (hypoesthesia) of the IAN after the coronectomy, which was recovered in its entirety at the 12 month mark of the follow-up. With regard to the eruption of the roots, it was observed in 3% of cases, with the most distant at the 24 month mark. All of them were re-operated, and no one developed any kind of alteration in the IAN. Finally, they estimated that the average maximum root migration was 2.9 mm at 24 months after the operation, which is greatest in the period between the 0 and 6 months (1.9 mm). They also estimated that 75.2% stopped migrating at the 12 - 24 month mark, and that subsequently there were no reactivations that were detected in these cases. Finally, in no case was the development of an apical infection in the retained roots observed (20).

(vi) Follow-up

In spite of the fact that the methodologies of the studies suggest the consistent follow-up at 7 days after surgery and at the 3, 6, 12 and 24 months, usually there were few studies that were able to carry out successful follow-up procedures with all the patients up until after the one year mark. Although 12 months are sufficient to assess some variables like injury to the IAN, postoperative infection, dry socket and pain, it is not enough for the analysis of the side effects of the root or roots present. Such side effects include: migration and exposure of the root, infection, or other possible long-term complications.

Leung *et al*. (20), in their study of prospective cohorts analyzed the long-term results of their original study from 2009. In 2009 in the randomized clinical trial (15) they treated 171 lower third molars with a coronectomy, with an average follow up of 10.6 months. In the current study of cohorts from 2012, the results were analyzed after a follow-up in 98 patients (135 coronectomies) up to the 36 month mark, making it of all of the study, the only one which did a long term follow to analyze their patients in the long term. However, we believe that the biggest limitation of Leung *et al*. (20) in their research work is that the selection of the sample is only based on the panoramic x-ray, which may significantly alter the obtained results, since in some cases they really might not be in direct contact with the inferior dental canal, and this is reflected in the study of Matzen *et al*. (21), where it was concluded that if a CBCT is performed on the sample, in 12% of the cases the treatment plan will be changed (from a coronectomy to a complete extraction) (21).

## Conclusions

Finally, after examining the literature review, we can conclude that the coronectomy is an adequate preventative technique in IAN protection. It is shown as an alternative to the conventional extraction of third molars in which there is a high risk of injury to the inferior alveolar nerve.

However, there is a need for new clinical studies, with a greater number of samples, with a randomized approach, and with a long follow-up period in order to detect the potential adverse effects of the retained roots. Additionally, these new clinical trials should incorporate variables that have not been previously analyzed, therefore highlighting the periodontal recovery of the second distal molar, the proper clinical evaluation of the IAN injury, as well as the correlation between the position of the lower third molar and failure of the coronectomy.

## References

[1] Long H, Zhou Y, Liao L, Pyakurel U, Wang Y, Lai W (2012). Coronectomy vs. total removal for third molar extraction: a systematic review. J Dent Res.

[2] Renton T, Hankins M, Sproate C, McGurk M (2005). A randomised controlled clinical trial to compare the incidence of injury to the inferior alveolar nerve as a result of coronectomy and removal of mandibular third molars. Br J Oral Maxillofac Surg.

[3] Robinson PP, Loescher AR, Yates JM, Smith KG (2004). Current management of damage to the inferior alveolar and lingual nerves as a result of removal of third molars. Br J Oral Maxillofac Surg.

[4] Renton T (2012). Notes on coronectomy. Br Dent J.

[5] Gleeson CF, Patel V, Kwok J, Sproat C (2012). Coronectomy practice. Paper 1. Technique and trouble-shooting. Br J Oral Maxillofac Surg.

[6] Patel V, Moore S, Sproat C (2010). Coronectomy – oral surgery's answer to modern day conservative dentistry. Br Dent J.

[7] O'Riordan BC (2004). Coronectomy (intentional partial odontectomy of lower third molars). Oral Surg Oral Med Oral Pathol Oral Radiol Endod.

[8] Céspedes-Sánchez JM, Ayuso-Montero R, Marí-Roig A, Arranz-Obispo C, López-López J (2014). The importance of a good evaluation in order to prevent oral nerve injuries: A review. Acta Odontol Scand.

[9] Sencimen M, Ortakoglu K, Aydin C, Aydintug YS, Ozyigit A, Ozen T (2010). Is endodontic treatment necessary during coronectomy procedure?. J Oral Maxillofac Surg.

[10] Ecuyer J, Debien J (1984). [Surgical deductions]. Actual Odontostomatol (Paris).

[11] Pogrel MA, Lee JS, Muff DF (2004). Coronectomy: a technique to protect the inferior alveolar nerve. J Oral Maxillofac Surg.

[12] Patel V, Gleeson CF, Kwok J, Sproat C (2013). Coronectomy practice. Paper 2: complications and long term management. Br J Oral Maxillofac Surg.

[13] Shekelle PG, Ortiz E, Rhodes S, Morton SC, Eccles MP, Grimshaw JM (2001). Validity of the Agency for Healthcare Research and Quality clinical practice guidelines: how quickly do guidelines become outdated?. JAMA.

[14] Jadad AR, Moore RA, Carroll D, Jenkinson C, Reynolds DJ, Gavaghan DJ (1996). Assessing the quality of reports of randomized clinical trials: is blinding necessary?. Control Clin Trials.

[15] Leung YY, Cheung LK (2009). Safety of coronectomy versus excision of wisdom teeth: a randomized controlled trial. Oral Surg Oral Med Oral Pathol Oral Radiol Endod.

[16] Hatano Y, Kurita K, Kuroiwa Y, Yuasa H, Ariji E (2009). Clinical evaluations of coronectomy (intentional partial odontectomy) form mandibular third molars using dental computed tomography: a case-control study. J Oral Maxillofac Surg.

[17] Cilasun U, Yildirim T, Guzeldemir E, Pektas ZO (2011). Coronectomy in patients with high risk of inferior alveolar nerve injury diagnosed by computed tomography. J Oral Maxillofac Surg.

[18] Goto S, Kurita K, Kuroiwa Y, Hatano Y, Kohara K, Izumi M (2012). Clinical and dental computed tomographic evaluation 1 year after coronectomy. J Oral Maxillofac Surg.

[19] Monaco G, de Santis G, Gatto MR, Corinaldesi G, Marchetti C (2012). Coronectomy: a surgical option for impacted third molars in close proximity to the inferior alveolar nerve. J Am Dent Assoc.

[20] Leung YY, Cheung LK (2012). Coronectomy of the lower third molar is safe within the first 3 years. J Oral Maxillofac Surg.

[21] Matzen LH, Christensen J, Hintze H, Schou S, Wenzel A (2013). Influence of cone beam CT on treatment plan before surgical intervention of mandibular third molars and impact of radiographic factors on deciding on coronectomy vs surgical removal. Dentomaxillofac Radiol.

[22] Dolanmaz D, Yildirim G, Isik K, Kucuk K, Ozturk A (2009). A preferable technique for protecting the inferior alveolar nerve: coronectomy. J Oral Maxillofac Surg.

